# Multiferroic/Polymer Flexible Structures Obtained by Atomic Layer Deposition

**DOI:** 10.3390/nano13010139

**Published:** 2022-12-27

**Authors:** Shikhgasan Ramazanov, Dinara Sobola, Gaji Gajiev, Farid Orudzhev, Pavel Kaspar, Adil Gummetov

**Affiliations:** 1 Amirkhanov Institute of Physics, Dagestan Federal Research Center, Russian Academy of Sciences, Makhachkala 367003, Russia; 2 Department of Physics, Faculty of Electrical Engineering and Communication, Brno University of Technology, Technicka 2848/8, 61600 Brno, Czech Republic

**Keywords:** BiFeO_3_, flexible substrate, functionalization, multiferroic, polyimide, atomic layer deposition

## Abstract

The paper considers how a film of bismuth ferrite BiFeO_3_ (BFO) is formed on a polymeric flexible polyimide substrate at low temperature ALD (250 °C). Two samples of BFO/Polyimide with different thicknesses (42 nm, 77 nm) were studied. As the thickness increases, a crystalline BFO phase with magnetic and electrical properties inherent to a multiferroic is observed. An increase in the film thickness promotes clustering. The competition between the magnetic and electrical subsystems creates an anomalous behavior of the magnetization at a temperature of 200 K. This property is probably related to the multiferroic/polymer interface. This paper explores the prerequisites for the low-temperature growth of BFO films on organic materials as promising structural components for flexible and quantum electronics.

## 1. Introduction

Recently, there has been a growing interest in multiferroics with a perovskite structure, which attract the attention of researchers with their magnetic and electrophysical properties in combination with other materials, as evidenced by numerous works [1,2,3,4,5,6,7,8]. BiFeO_3_ (BFO) has high magnetic (T_N_ = 643 K) and ferroelectric (T_C_ = 1103 K) ordering temperatures compared to other multiferroics. Unlike thin films, the problem of bulk materials is the suppression of the magnetoelectric effect. Below the Neel temperature, BFO is a G-type antiferromagnet. A giant spontaneous polarization was also observed in BFO films [3]. Size effects at the grain boundaries and in films at the boundary with the substrate also create an additional intermediate antipolar phase in BFO, which affects the microscopic mechanisms of current flow [9]. The nature of the influence of this phase on the transport properties is still unknown. 

At the same time, fields of creation of magnetic polymers are rapidly developing. Magnetic polymers are functional polymers with magnetic properties, including paramagnetic, ferromagnetic, and ferrimagnetic ones [10,11]. Attempts have been made to create a polymer magnet from an organic polymer with both magnetic and superconducting properties. This issue still remains unresolved. In a centrosymmetric organic superconductor, there is a polarized current induced by a nonlinear charge acceleration, which is amplified by charge fluctuations. This can lead to the generation of higher harmonics and photoelectron emission [12]. However, the magnetic properties of the material were not investigated in this work. Magnetic polymers fall into three main categories: (1) purely organic magnetic polyradical molecules, (2) association of polymers with metal ions, and (3) embodiments of ferromagnetic particles based on polymer-composite/metal oxide [13]. Polymers are increasingly used in modern processing technology [14,15]. One of the widely used polymers in combination with other materials is a piezopolymer—polyvinylidene [16,17,18] and a conductive semiconductor—polyaniline [19,20]. The use of polyimide is an extension of the area of research utilizing of multiferroics in combination with a polymeric material. The development will make it possible to obtain economical and flexible materials with controlled adaptive properties. Moreover, certain advantages are provided by the use of polymeric materials as a means of modifying the properties of a multiferroic and the creation of other hybrid materials. For example, it makes it possible to combine the outstanding electromagnetic properties of a multiferroic with the advantageous mechanical characteristics of polymeric materials, such as flexibility. Due to the flexibility of the substrate, it is possible to additionally influence the properties of the multiferroic by external influences (mechanical, magnetic, and electric fields). Because of less physical effort required, it is possible to study dynamic effects. This is due to size effects in BFO thin films and ceramics [21,22,23]. Like most polymers, polyimide is able to interact with atomic or ionized oxygen [24]. An existing research [25] reported the use of silica films which act as a protective layer to prevent degradation of the polyimide film. However, in our particular case, the ability to interact with oxygen is of decisive importance for the formation of the film–substrate interface. In general, it can be stated that polyimide is a suitable choice for use as an active material substrate due to its optimal mechanical, electrical, chemical and thermal properties [26]. Another application of polyimide is as an absorber for irradiation in the (terahertz) THz range [27].

To obtain high-quality BFO layers, it is necessary to anneal it at temperatures above 325–500 °C, which in our case can damage the substrate. Thus, the realization of obtaining a high-quality BFO layer is confirmed by the effect on crystallization of both temperature and irradiation at lower temperatures [28,29]. In addition, the paper [30] provides an analysis of the influence of the methods and conditions of synthesis on the possibility of self-organization of restrictions on the growth of formed particles of solid phases during chemical reactions. As has been observed, nucleation is associated with a chemical reaction on the surface. As noted by the authors of the papers [31], in addition to other features, crystallization can also be associated with internal friction of a partially amorphous BFO film. The question of the influence of the substrate polymerization on the BFO film growth mechanism remains open [32]. Thus, during the atomic layer deposition (ALD) process, the BFO crystalline phase can also form at lower growth temperatures. The purpose of this work is to study the material of a flexible BFO/polymers composite with multiferroic properties synthesized by a low-temperature method.

## 2. Materials and Methods

Polyimide (poly(4,4′-oxydiphenylenepyromellitimide)) pieces 1 × 1 cm^2^ in size, chemical formula C_22_H_10_N_2_O_5_ (commercial name is Kapton, DuPont, DE, USA), were used as a substrate. This compound is stable over a wide temperature range from −273 to +400 °C. Thus, the material is suitable as a substrate at the selected growth temperature, where the most suitable temperature window in the ALD process is located [33]. Films of the BFO composition were obtained on an ML-200 setup (ALDCERAM, Boulder, CO, USA). The source of the bismuth precursor was (Tris(1-methoxy-2-methyl-2-propoxy)bismuth) short name Bi(mmp)_3_. Ferrocene (Fe(C_5_H_5_)_2_ was the source of iron. In the time interval between the launch of the precursors, the chamber was supplied with O_3_ for 5 s. Purge was carried out with N_2_, purity 5.0, purge time was 15 s. The evaporation temperature of Bi(mmp)_3_ was approximately 142 °C. The optimum temperature for evaporation of the ferrocene precursor was 87 °C. The chamber was uniformly heated up to 250 °C. The outlet gas was maintained at a constant temperature of 150 °C. The number of cycles for Sample 1 was 400, and for sample 2 cycles 600 (Table 1). Without functional groups on the surface of the substrate, it is impossible to provide a uniform coating of the BFO film, where the polyimide substrate is inert. However, the use of O_3_ during growth preliminarily oxidizes the surface and functionalizes it, which was noted during ALD Bi-Fe-O on the surface of highly oriented pyrolytic graphite (HOPG), as well as on the inhomogeneous surface of TiO_2_ (Nt) nanotubes in the works [34,35,36]. In this work, the research part was extended with different film thicknesses of the growth of the BFO phase and the self-assembly of a multiferroic film on a polyimide substrate was confirmed.

Raman measurements to analyze the vibrational modes of chemical bonds were performed on WITec alpha 300R device (WITec Wissenschaftliche Instrumente und Technologie GmbH, Ulm, Germany). Then, the X-ray photoelectron spectroscopy was carried out (XPS) AXIS SupraTM (Kratos Analytical Ltd, Manchester, UK), emission current: 15 mA, charge neutralizer: filament current 0.45 A, filament bias: 1.05 V, charge balance 4.6 V. For investigation of magnetic properties, the vibration magnetometer (Cryo-gen-Free High Field Measurement System from Cryogenic Limited, London, UK) was used. Scanning of the surface of the samples was carried out by the methods described in the works [37,38]. 

Electrical measurements were performed on the source-measurement device Keithley 2400 (Tektronix, Inc., Beaverton, OR, USA). The voltage sweep was carried out in the form of a bidirectional signal of a triangular shape (0→V→0→−V→0). At each sweep point, the measurement was carried out according to the method: voltage generation - delay (waiting) - measurement. The delay value varied from 0.01 s to 2 s. The instrument’s normal rate of measurement option was (was) selected. The pyroelectric current as a function of temperature was measured by the conventional two-probe method with Pt clamping electrodes; the temperature was raised at a rate of 15 s/°C. The P(U) dependence pattern was recorded on a Tektronics MSO-4034S oscilloscope. (Tektronix, Inc., Beaverton, OR, USA).

## 3. Results

**Figure 1:**
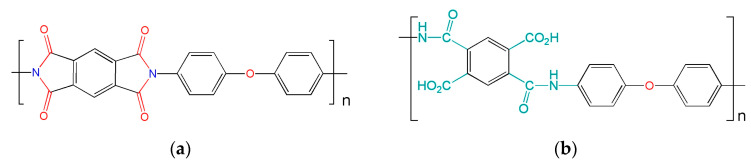
(**a**) Structural diagram of a polyimide molecule; (**b**) the most probable pattern of surface bond formation after the influence of ozone and heating temperature.

Oxidative degradation is more common in high temperature environments, so an alternative atmosphere is required to prevent a rapid reduction in film lifetime [39]. The probable structural components of the surface area of the substrate after the influence of ozone and temperature are shown in Figure 1, then the process of decomposition of the precursors and the formation of Bi-O and Fe-O bonds take place [40]. Inert surfaces are modified in various ways by ultrasonication with UV ozone or KOH, this mechanism has also been tested on an inert HOPG (highly oriented pyrolytic graphite) surface [34]. In the ALD process, in addition to the ozone oxidizer, the substrate temperature of 250 °C additionally accelerates the surface destruction reaction. Figure 1 shows the primary structure of the polyimide before and after surface bonding.

The effect of O_3_ and temperature creates hydroxyl, carboxyl, and amine groups (HO_2_C, CO_2_H, NH, OH) on the polyimide surface, which interact with organometallic precursors in subsequent precursor injection cycles. Table 1 shows the parameters and designations of the obtained samples by the ALD method.

**Figure 2:**
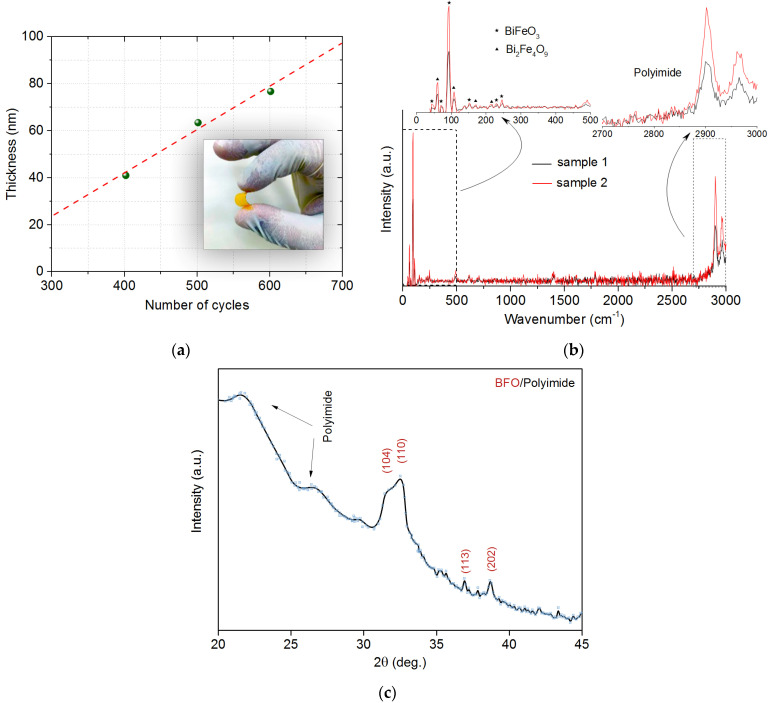
(**a**) Change in film thickness during growth depending on the number of cycles. (**b**) Raman spectrum from the obtained sample BFO/polyimide. (**c**) XRD from sample 2 BFO/polyimide.

Figure 2a shows the change in film thickness during growth as a function of the number of cycles. The dependence is almost linear at small thicknesses. Figure 2b shows the Raman spectrum, where the initial part of the spectrum from the polyimide substrate is quenched by the film, and then peaks from the substrate are revealed in the region of 3000 cm^−1^. Figure 2c shows X-ray diffraction analysis (XRD) of BFO/polyimide Sample 2. For the first sample, the background from the polymer substrate covers all peaks due to the low film thickness.

**Figure 3:**
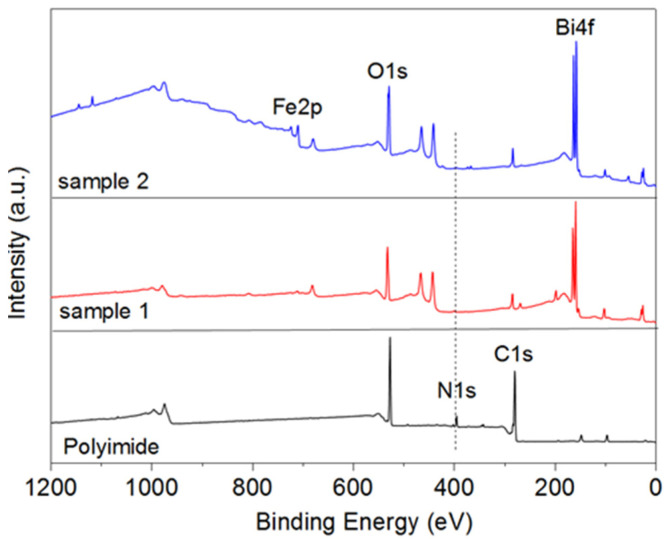
XPS spectrum of BFO/polyimide samples. The spectra of a clean substrate are shown, as well as how the spectrum changes at different thicknesses.

The Raman spectrum shows that the intensity of a thicker sample is higher than that of a thin one. Functionalization by bismuth occurs in the resulting organometallic binder and charge transfer occurs, a similar peak in the region of 1400 cm^−1^ is visible on the Raman spectrum. An ionic bond with Bi^3+^ and Bi(NO_3_)_3_ can also be formed [41]. During ALD growth, partial segregation of Bi atoms from the surface region of the substrate was observed during the oxidation of the substrate [25]. The N atoms remain in bond as well as C and O (Figure 3). The width and intensity of the peaks of the Raman spectrum (Figure 2b) correspond to the form of oxidation of bismuth. It was also possible to observe photoluminescence, which is a consequence of the partial self-organization of Bi-O as a result of laser irradiation and the presence of oxygen vacancies. As expected, it was higher in sample 2; for peak comparison, the photoluminescence contribution was subtracted as a background. As can be seen in the tab of Figure 2b, the Raman displacement in the Bi-Fe-O system has a main and a parasitic phase of selenite (Bi_2_Fe_4_O_9_). The main acoustic and optical modes relevant for BFO are 43 cm^−1^, 70 cm^−1^, 93 cm^−1^, 154 cm^−1^, 228 cm^−1^, and 245 cm^−1^. Structural study of the phase composition on XRD showed the corresponding phases where the main peaks (104), (110) corresponding to the rhombohedral R3c lattice are present. There is also a partially parasitic phase of the compound Bi_2_Fe_4_O_9_. The appearance of other peaks (113), (202) from the BFO film showed polycrystalline structure.

As can be seen on XPS, the small thickness does not allow FeO_x_ to crystallize, the crystalline phase is formed in conjunction with Bi-O during growth and over the course of a chemical reaction [40]. The additional peaks that appeared for the BFO film in the region of 420 eV are related to 4d electrons with spin orbital splitting 3/2 and 5/2.

## 4. Discussion

XPS suggests that in the second case, bismuth is less oxidized and self-organizes, agglomerating into partially metallic inclusions (Figure 3). The second sample has more dangling oxygen bonds, which means the material is more amorphous. This effect creates an excess of oxygen. The consequence of excess oxygen on the sample is the broadening of the iron peak. The Fe–O and Bi–O phases are also formed in Sample 1. This is a more favorable combination for the subsequent formation of the BFO phase. The figure shows the dynamics of changes in individual parts of the spectrum related to C1s, O1s, and N1s electrons. These spectra are more related to the intermediate film-substrate phase than to the main BFO film (if we do not take into account oxygen, which is part of both the polymer and the film).

**Figure 4:**
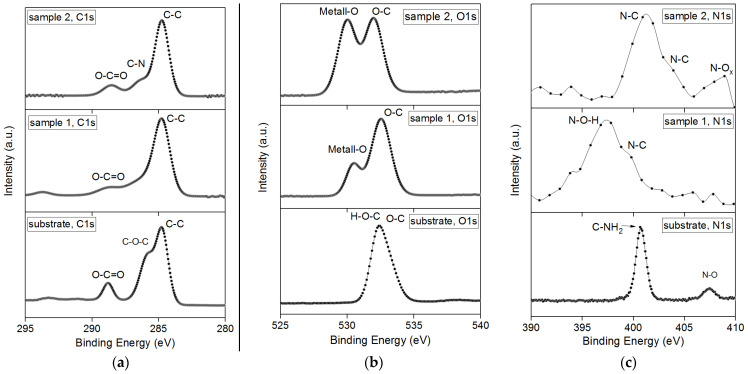
XPS from samples and pure polyimide prior to film deposition: (**a**) XPS from 1s carbon electrons; (**b**) XPS from 1s oxygen electrons; (**c**) XPS from nitrogen 1s electrons.

Figure 4 shows the spectra related to the process of modifying the near-surface region of the polyimide substrate. As can be seen from the 1s electronic states for carbon in sample 1, surface modification occurs, but metal bonds with oxygen are formed (Figure 4b) and many nitrogen groups remain in the film. Furthermore, with an increase in the thickness of the BFO film, an increase in metal bonds (sample 2 O1s) occurs and, accordingly, the nitrogen spectrum also equalizes. The broadening indicates the interaction of the near-surface region of the film-substrate (adhesion improves). The shift of intense peaks for N1s (Figure 4c) electrons is probably due to the formation of an intermediate phase between the film and the substrate.

**Figure 5:**
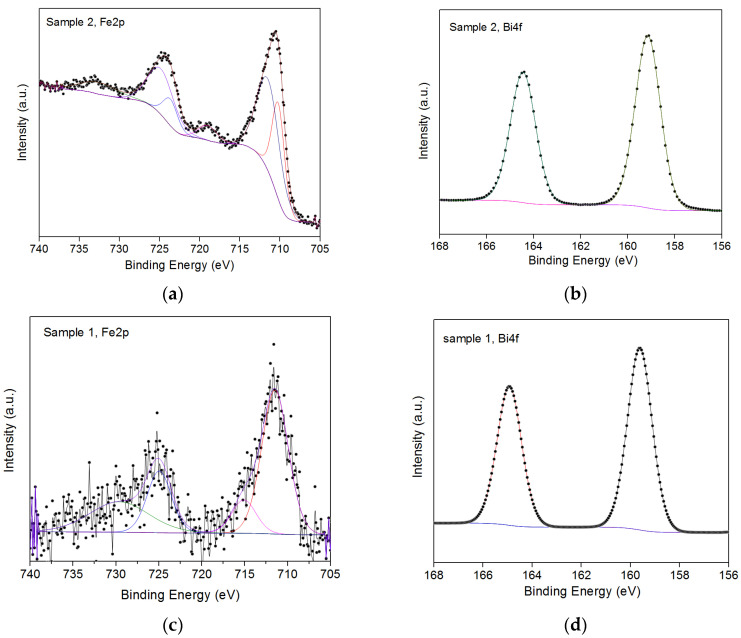
XPS from samples related to BFO film composition: (**a**,**c**) XPS from iron 2p electrons; (**b**,**d**) XPS from bismuth 4f electrons.

The following Figure 5 shows the portions of the spectrum related to the BFO phase.

As can be seen from Figure 5, for iron, the ratio of low-spin and high-spin is different, where 2p_3/2_ is quantitatively greater than 2p_1/2_, and also a small spin-orbit splitting is not so developed, which is associated with the partial amorphousness of the sample 1 thin film (Figure 5c). In sample 2 (Figure 5a), the film phase already crystallizes. The increase in thickness in accordance with the Gaussian distribution shown for iron electrons indicates an increase in the composition of Fe^2+^ over Fe^3+^ ions, which is characteristic of the BFO phase. For 4f electrons, the intensity is practically the same for the two samples. A slight shift of the peaks can be observed for Bi4f_7/2_ sample 1 (159.7 eV) relative to sample 2 (159.2 eV) by 0.5 eV. The spin–orbit splitting of 4f electrons for both samples was 5.2 eV ± 0.1 eV. During the synthesis of the film, a Bi_2_O_3_ crystalline phase was formed. A further change in the film thickness in the Bi-Fe-O system contributes to the redistribution of oxygen between the iron oxide phases. 

**Figure 6:**
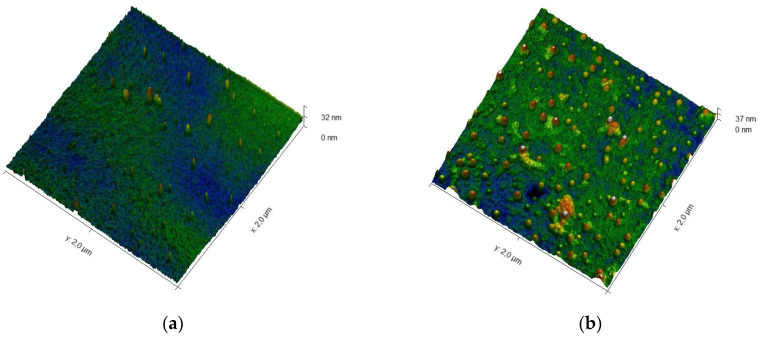
AFM of the surface area of BFO/Polyimide samples: (**a**) sample 1, surface morphology is not pronounced; (**b**) Sample 2, the crystalline phase is self-organized in the film.

Additionally, the crystallization of the near-surface region was studied by probe methods. The film growth morphology changes due to an increase in the film thickness. The Figure 6 shows the spatial patterns of the AFM surface. As can be seen in Figure 6, partial coalescence occurs in the sample. As the surface morphology grows, the features of the interaction of these clusters can create new properties. The calculation of the fractal dimension showed fractal dimension 2.460, for sample 2 fractal dimension 2.564. The increase in fractality with increasing BFO film thickness confirms the mixed film growth mechanism [42].

**Figure 7:**
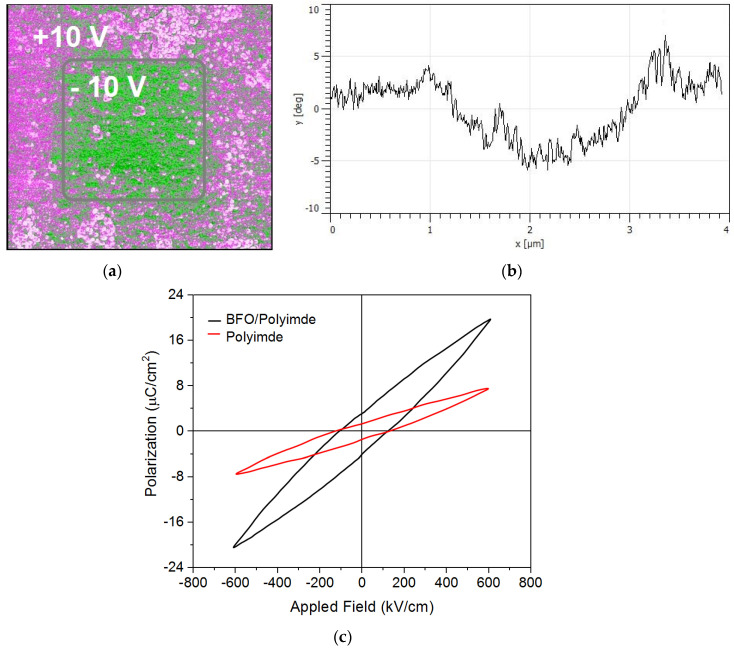
(**a**) PFM area 4 × 4 µm^2^ of BFO film surface of sample 2; (**b**) phase change along the line; (**c**) polarization-electric field (D–E) hysteresis loop curve for pure polymer and BFO/polyimide composite at 1 kHz.

Elevated areas 30–50 nm in size similar to quantum dots are formed on the film surface. This feature may be important in the future, since such dotted areas are self-organized. Piezoresponse force microscopy (PFM) was used to investigate the possibility of switching the ferroelectric polarization [43]. Figure 7 shows a scan of the film surface area of sample 2, as well as the phase change along the line. Figure 7c shows the polarization-electric field (D–E) hysteresis loop curve for pure polymer and BFO/polyimide composite alone at frequency of 1 kHz. The measurement of the dielectric hysteresis loop was carried out according to the Sawyer–Tower method. An external voltage signal formed on an Agilent Technologies generator in the form of a bidirectional triangle with an amplitude of ±15 V and a duration of 1.5 periods was applied to the structure.

**Figure 8:**
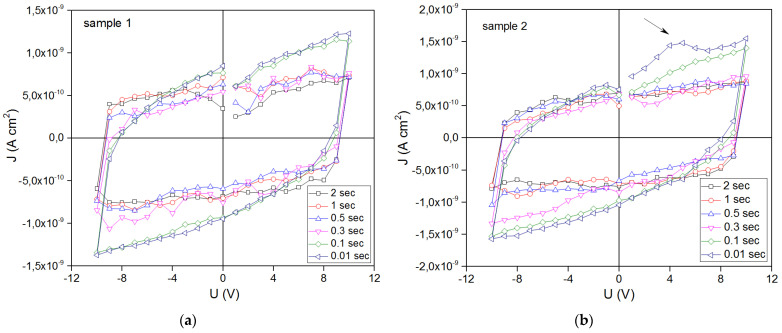
Current density vs applied voltage of a BFO/polyimide samples at different delay-measurement times: (**a**) sample 1, linear change; (**b**) sample 2, the appearance of a non-linear section at the time of delay measurement from t = 0.1 s.

In the study of ferroelectric switching by the PFM method, no changes were revealed in the first sample. This is probably due to the weakly pronounced electrical parameters of the thin film. On sample 2, when a voltage of different polarity ± 10 V was applied, the phase changed (Figure 7). These characteristics confirm the mechanism of ferroelectric switching. The dielectric constant of polyimide films is typically between 2.78 and 3.48 [44] and dielectric strength is from 150 V/µm to 300 V/µm at standard temperature (23 °C) and relative humidity (50%). Frequency, on the other hand, has little to no effect on the dielectric constant of the material, even though increasing frequency causes a small increase in dissipation factor [45]. The study of the ferroelectric properties of the samples showed an increase in polarization for samples with a BFO film. As can be seen in Figure 7c, the parameters of dielectric constant, polarization, and breakdown strength are directly related to the stored energy in dielectric materials and can increase the energy density. The influence of the polyimide substrate on the electrical properties is negligible. The current density versus applied voltage for the two samples is shown in Figure 8.

**Figure 9:**
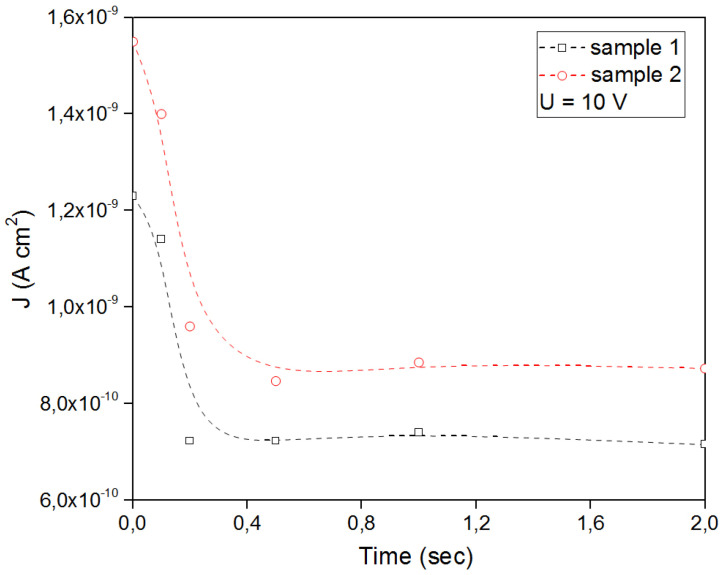
Dependence of the current density at 10 V applied voltage at different measurement frequencies on t = 0.01 ÷ 2 s.

As can be seen in Figure 8, the polarization of sample 2 is greater. There is an increase in polarization and an increase in the angle of rotation of the loop at t = 0.1 ÷ 0.01 s, which indicates a quasi-dynamic mode of the dependence of the current density. In Figure 8b for sample 2 in the 5 V region, another “hump” is seen showing a nonlinear contribution at t = 0.01 s. Perhaps this is indirectly related to the magnetization of the sample, since it is greater in the second sample. Next, in Figure 9, we considered this quasi-dynamic mechanism of current density versus applied voltage and plotted the dependence for the region with maximum polarizations at 10 V. Changing the time between applying voltage and recording the current density affects the mechanism of current flow in such structures. As can be seen in Figure 9, at t < 0.1 s, a nonlinear section J and then an almost linear section begins from t = 0.2 s. This feature is probably related to the mechanisms of charge capture by defects, as we know they are partially ionized and can affect conduction electrons. The ferroelectric nature of BFO can improve carrier separation [46]. In classical electrodynamics, the volume integral J is the time derivative of the electric dipole moment of the system:

(1) $$\int {\mathrm{Jd}}^{3}V=\underset{i}{\sum }\int J_{i}d^{3}=\underset{i}{\sum }q_{i}v_{i}=\underset{i}{\sum }q_{i}\dot{x_{i}}=\;\dot{d}$$

This is approximately the product of current and displacement between the centers of positive and negative charges, namely:

(2) $$\dot{d}\sim \;I∆l$$

In the quasi-dynamic measurement mode at small t, between applying the voltage and recording the current density, a nonlinear dependence J(t) of charge separation is formed, probably due to the fact that the energy of the conduction electron partially retains its charge without having time to scatter on the phonons of the crystal lattice. Ionization of defect centers act as Coulomb traps for mobile electrons, which contributes to an increase in the local density of states [47]. Discharged areas decrease the rate of junctions. The regions of micromagnetism arising as a result of low dimensionality affect the general polarization properties of the sample [48].

**Figure 10:**
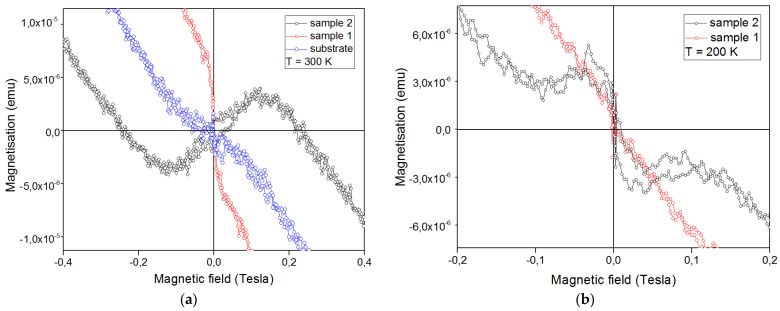
Magnetic hysteresis loops M (H) for BFO/polyimide samples at (**a**) 300 K and (**b**) 200 K.

There is magnetoelectric coupling in BFO, especially in films, expressed due to anisotropy [49]. To understand the magnetic properties of the obtained films and relate them to the non-linearity of the current flow in the sample, Figure 10 shows magnetic hysteresis loops. The contribution from the substrate has not been subtracted but is shown separately in the graph of Figure 10a (blue line). Measurements of M (H) were carried out at temperatures of 300 K and 200 K. Since the dimensions of the samples were 1.0 cm^2^, the values along the y-axis can be referred to emu/cm^2^.

Taking into account the volume of the film thickness for sample *2* M_max_ = 0.31 emu/cc, this value of magnetization is practically comparable with the BFO antiferromagnetic ceramics [49]. The appearance of magnetic hysteresis at room temperature indicates the antiferromagnetic properties of the obtained films (Figure 10). The paramagnetic component is also seen, the substrate exhibits the properties of a diamagnet as seen in Figure 10a. A ferromagnetic phase associated with Fe − Fe exchange interactions already appear in sample 2, the low magnetization (4.6 × 10^−6^ emu) can be attributed to the ferrimagnetic nature of the film. An interesting feature was noticed when the measurement temperature decreased by 100 K from room temperature (300 K). The non-standard behavior of the magnetization of the multiferroic layer is possibly due to the influence of organics on the film, as was shown in the work [50]. As T decreases, sample 1 completely transforms into the paramagnetic phase; due to AFM/PM competition, the PM phase predominates. For sample 2, it reverses and the PM phase cannot benefit from the already-in-place crystallinity of the film, but the rotational contribution still remains similar to the micromagnetism phase.

**Figure 11:**
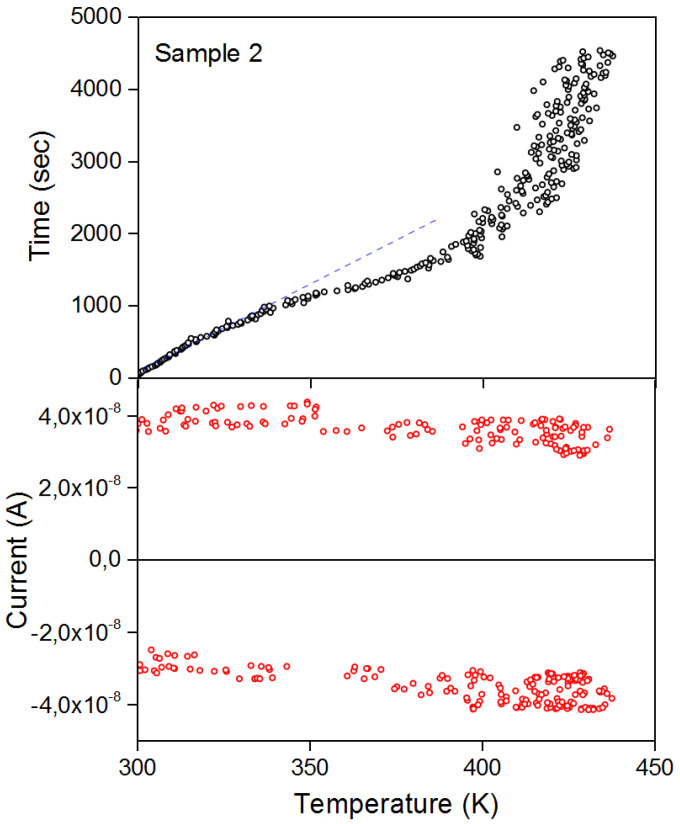
Pyroelectric current sample 2 BFO/Polyimide. The top graph shows the change in current over time.

Changes in the temperature of polyimide are known to cause smaller changes in electrical properties, although not entirely negligible. Taking into account the conducted studies, we decided to additionally look at the properties of the pyroelectric current (Figure 11).

To see the nonlinear part of I(T), we increase the perturbation of the crystal lattice; probably, with increasing temperature, the scattering coefficient on ionized traps will increase. On sample 1, a change in the pyroelectric current was not noticed, perhaps this is due to the small thickness of the film as well as high values of the dielectric constant. Thus, we investigated the highest quality sample 2 exhibiting both electrical and magnetic properties. As can be seen from Figure 11, at the initial stage of heating, the pyrocurrent has a linear section, this can be seen from the temporal characteristic of the pyrocurrent (blue dashed line on the graph). In the structure of the film, there is an amorphous and crystalline phase, this is confirmed by the current transport mechanisms in the studied sample. As you can see, pyrolysis changes its character with increasing temperature, so it can be assumed that over time and during heating of the carriers (scattering by ionized impurities), a charge transfer occurs and the potential well changes. The magnetization (Figure 10b) shows that as the temperature decreases, the scattering on ionized impurities decreases. This perhaps shows a tandem effect.

## 5. Conclusions

This study highlights the relevance and importance of using compliant Kapton substrates for designing microstructures in the field of electronics. The properties of the films obtained by the ALD method on a polyimide polymer film were demonstrated. XPS and Raman spectroscopy methods were used to provide a structural and compositional characterization of the resulting layers. An analysis of the surface topography showed the process of self-organization of the film and the formation of clusters on the surface. Electrical and magnetic measurements showed the multiferroicity of the resulting film. Some features associated with the thickness of the BFO film are noted. The results described in this study are useful for improving the reproducibility of the discussed structures, as well as the physical properties of the multiferroic material on the surface of the polymer substrate.

## Figures and Tables

**Table 1:** Characteristics and designations of the obtained samples BFO/Polyimide.

Sample	Number of Cycles	Film Thickness
Sample 1	400	42
Sample 2	600	77

## Data Availability

The data that support the findings of this study are available from the corresponding authors upon reasonable request.

## References

[1] Shimizu K., Kawabe R., Hojo H., Shimizu H., Yamamoto H., Katsumata M., Shigematsu K., Mibu K., Kumagai Y., Oba F. (2019). Direct Observation of Magnetization Reversal by Electric Field at Room Temperature in Co-Substituted Bismuth Ferrite Thin Film. Nano Lett..

[2] An H., Hong H.J., Jo Y.-R., Jung S.-G., Kim S., Kim S., Lee J., Choi H., Yoon H., Kim S.-Y. (2019). Reversible magnetoelectric switching in multiferroic three-dimensional nanocup heterostructure films. NPG Asia Mater..

[3] Rossell M.D., Erni R., Prange M.P., Idrobo J.-C., Luo W., Zeches R.J., Pantelides S.T., Ramesh R. (2012). Atomic Structure of Highly Strained BiFeO_3_Thin Films. Phys. Rev. Lett..

[4] Markelova M., Nygaard R., Tsymbarenko D., Shurkina A., Abramov A., Amelichev V., Makarevich A., Vasiliev A., Kaul A. (2021). Multiferroic h-LuFeO 3 Thin Films on (111) and (100) Surfaces of YSZ Substrates: An Experimental and Theoretical Study. ACS Appl. Electron. Mater..

[5] Goswami S., Dey K., Chakraborty S., Giri S., Chowdhury U., Bhattacharya D. (2020). Large Magnetoelectric Coupling in the Thin Film of Multiferroic CuO. ACS Omega.

[6] Kumar M., Shankar S., Kumar A., Anshul A., Jayasimhadri M., Thakur O.P. (2020). Progress in multiferroic and magnetoelectric materials: Applications, opportunities and challenges. J. Mater. Sci. Mater. Electron..

[7] Hojo H., Kawabe R., Shimizu K., Yamamoto H., Mibu K., Samanta K., Saha-Dasgupta T., Azuma M. (2017). Ferromagnetism at Room Temperature Induced by Spin Structure Change in BiFe_1−x_Co_x_O_3_ Thin Films. Adv. Mater..

[8] Jana B., Ghosh K., Rudrapal K., Gaur P., Shihabudeen P.K., Roy Chaudhuri A. (2022). Recent Progress in Flexible Multiferroics. Front. Phys..

[9] Liu B., Yang C., Li X., Wang C., Liu G., Yang H., Wang Y. (2018). Origin of antipolar clusters in BiFeO_3_ epitaxial thin films. J. Eur. Ceram. Soc..

[10] Lu Q., Choi K., Nam J.-D., Choi H.J. (2021). Magnetic Polymer Composite Particles: Design and Magnetorheology. Polymers.

[11] Sorokin V.V., Stepanov G.V., Shamonin M., Monkman G.J., Khokhlov A.R., Kramarenko E.Y. (2015). Hysteresis of the viscoelastic properties and the normal force in magnetically and mechanically soft magnetoactive elastomers: Effects of filler composition, strain amplitude and magnetic field. Polymer.

[12] Kawakami Y., Amano T., Ohashi H., Itoh H., Nakamura Y., Kishida H., Sasaki T., Kawaguchi G., Yamamoto H.M., Yamamoto K. (2020). Petahertz non-linear current in a centrosymmetric organic superconductor. Nat. Commun..

[13] Salem S., Yilmaz E. (2021). Magnetic nanoparticle-polymer hybrid materials. Magnetic Nanoparticle-Based Hybrid Materials.

[14] Kaspar P., Sobola D., Částková K., Knápek A., Burda D., Orudzhev F., Dallaev R., Tofel P., Trčka T., Grmela L. (2020). Characterization of Polyvinylidene Fluoride (PVDF) Electrospun Fibers Doped by Carbon Flakes. Polymers.

[15] Castkova K., Kastyl J., Sobola D., Petrus J., Stastna E., Riha D., Tofel P. (2020). Structure–Properties Relationship of Electrospun PVDF Fibers. Nanomaterials.

[16] Giannelli P., Bulletti A., Capineri L. (2017). Multifunctional Piezopolymer Film Transducer for Structural Health Monitoring Applications. IEEE Sens. J..

[17] Wang Y., Ren K., Zhang Q.M. (2007). Direct piezoelectric response of piezopolymer polyvinylidene fluoride under high mechanical strain and stress. Appl. Phys. Lett..

[18] Orudzhev F., Ramazanov S., Sobola D., Kaspar P., Trčka T., Částková K., Kastyl J., Zvereva I., Wang C., Selimov D. (2021). Ultrasound and water flow driven piezophototronic effect in self-polarized flexible α-Fe_2_O_3_ containing PVDF nanofibers film for enhanced catalytic oxidation. Nano Energy.

[19] Yakuphanoglu F., Şenkal B.F. (2007). Electronic and Thermoelectric Properties of Polyaniline Organic Semiconductor and Electrical Characterization of Al/PANI MIS Diode. J. Phys. Chem. C.

[20] Mocioiu A.-M., Tudor I.A., Mocioiu O.C. (2021). Application of Polyaniline for Flexible Semiconductors. Coatings.

[21] Wang N., Luo X., Han L., Zhang Z., Zhang R., Olin H., Yang Y. (2020). Structure, Performance, and Application of BiFeO_3_ Nanomaterials. Nano-Micro Lett..

[22] Alikhanov N.M.-R., Rabadanov M.K., Orudzhev F.F., Gadzhimagomedov S.K., Emirov R.M., Sadykov S.A., Kallaev S.N., Ramazanov S.M., Abdulvakhidov K.G., Sobola D. (2021). Size-dependent structural parameters, optical, and magnetic properties of facile synthesized pure-phase BiFeO3. J. Mater. Sci. Mater. Electron..

[23] Park T.-J., Papaefthymiou G.C., Viescas A.J., Moodenbaugh A.R., Wong S.S. (2007). Size-Dependent Magnetic Properties of Single-Crystalline Multiferroic BiFeO 3 Nanoparticles. Nano Lett..

[24] Chandra Das S., Majumdar A., Katiyal S., Poojitha B., Saha S., Shripathi T. (2017). Phase pure epitaxial growth of BiFeO_3_ films: An effect of oxygen partial pressure. Solid State Commun..

[25] Sobola D., Ramazanov S., Konečný M., Orudzhev F., Kaspar P., Papež N., Knápek A., Potoček M. (2020). Complementary SEM-AFM of Swelling Bi-Fe-O Film on HOPG Substrate. Materials.

[26] Signore M.A., Taurino A., Catalano M., Kim M., Che Z., Quaranta F., Siciliano P. (2017). Growth assessment of (002)-oriented AlN thin films on Ti bottom electrode deposited on silicon and kapton substrates. Mater. Des..

[27] Zhai D., Yang Y., Geng Z., Cui B., Zhao R. (2018). A High-selectivity THz Filter Based on A Flexible Polyimide Film. IEEE Trans. Terahertz Sci. Technol..

[28] Bretos I., Jiménez R., Ricote J., Sirera R., Calzada M.L. (2020). Photoferroelectric Thin Films for Flexible Systems by a Three-in-One Solution-Based Approach. Adv. Funct. Mater..

[29] Li Z., Wang Z.L., Wang Z. (2018). In situ tuning of crystallization pathways by electron beam irradiation and heating in amorphous bismuth ferrite films. RSC Adv..

[30] Almjasheva O.V., Popkov V.I., Proskurina O.V., Gusarov V.V. (2022). Phase formation under conditions of self-organization of particle growth restrictions in the reaction system. Nanosyst. Phys. Chem. Math..

[31] Gridnev S.A., Kalinin Y.E., Dybov V.A., Popov I.I., Kashirin M.A., Tolstykh N.A. (2022). Internal friction in thin-film ferrite bismuth with an amorphous structure. J. Alloys Compd..

[32] Catalan G., Scott J.F. (2009). Physics and applications of bismuth ferrite. Adv. Mater..

[33] Marchand B., Jalkanen P., Tuboltsev V., Vehkamäki M., Puttaswamy M., Kemell M., Mizohata K., Hatanpää T., Savin A., Räisänen J. (2016). Electric and Magnetic Properties of ALD-Grown BiFeO 3 Films. J. Phys. Chem. C.

[34] Ramazanov S., Sobola D., Orudzhev F., Knápek A., Polčák J., Potoček M., Kaspar P., Dallaev R. (2020). Surface modification and enhancement of ferromagnetism in BiFeO_3_ nanofilms deposited on HOPG. Nanomaterials.

[35] Orudzhev F., Ramazanov S., Sobola D., Isaev A., Wang C., Magomedova A., Kadiev M., Kaviyarasu K. (2020). Atomic layer deposition of mixed-layered aurivillius phase on TiO_2_ nanotubes: Synthesis, characterization and photoelectrocatalytic properties. Nanomaterials.

[36] Orudzhev F.F., Ramazanov S.M., Isaev A.B., Alikhanov N.M.-R., Sobola D., Presniakov M.Y., Kaviyarasu K. (2021). Self-organization of layered perovskites on TiO_2_ nanotubes surface by atomic layer deposition. Mater. Today Proc..

[37] Knápek A., Dallaev R., Burda D., Sobola D., Allaham M.M., Horáček M., Kaspar P., Matějka M., Mousa M.S. (2020). Field Emission Properties of Polymer Graphite Tips Prepared by Membrane Electrochemical Etching. Nanomaterials.

[38] Knápek A., Sýkora J., Chlumská J., Sobola D. (2017). Programmable set-up for electrochemical preparation of STM tips and ultra-sharp field emission cathodes. Microelectron. Eng..

[39] Tuttle J., DiPirro M., Canavan E., Hait T., Balachandran U., Amm K., Evans D., Gregory E., Lee P., Osofsky M. (2008). Thermal properties of double-aluminized kapton at low temperatures. AIP Conf. Proc..

[40] Ramazanov S., Sobola D., Ţălu Ş., Orudzev F., Arman A., Kaspar P., Dallaev R., Ramazanov G. (2022). Multiferroic behavior of the functionalized surface of a flexible substrate by deposition of Bi_2_O_3_ and Fe_2_O_3_. Microsc. Res. Tech..

[41] Perla V.K., Ghosh S.K., Mallick K. (2019). Nonvolatile switchable resistive behaviour via organic–inorganic hybrid interactions. J. Mater. Sci..

[42] Ţălu Ş., Stach S., Ramazanov S., Sobola D., Ramazanov G. (2017). Multifractal characterization of epitaxial silicon carbide on silicon. Mater. Sci..

[43] Zhang Q., Rana A., Liu X., Valanoor N. (2019). Electrode Dependence of Local Electrical Properties of Chemical-Solution-Deposition-Derived BiFeO 3 Thin Films. ACS Appl. Electron. Mater..

[44] Chisca S., Sava I., Musteata V.-E., Bruma M. Dielectric and conduction properties of polyimide films. Proceedings of the CAS 2011 Proceedings (2011 International Semiconductor Conference).

[45] He J.-J., Yang H.-X., Zheng F., Yang S.-Y. (2022). Dielectric Properties of Fluorinated Aromatic Polyimide Films with Rigid Polymer Backbones. Polymers.

[46] Sun Y., Sun Z., Wei R., Huang Y., Wang L., Leng J., Xiang P., Lan M. (2018). First principles study of the magnetic properties and charge transfer of Ni-doped BiFeO_3_. J. Magn. Magn. Mater..

[47] Yoo S.-J., Kim J.-J. (2015). Charge Transport in Electrically Doped Amorphous Organic Semiconductors. Macromol. Rapid Commun..

[48] Pyatakov A.P., Sergeev A.S., Nikolaeva E.P., Kosykh T.B., Nikolaev A.V., Zvezdin K.A., Zvezdin A.K. (2015). Micromagnetism and topological defects in magnetoelectric media. Physics.

[49] Orudzhev F.F., Ramazanov S.M., Sobola D., Alikhanov N.M.R., Dallaev R.S., Kasinathan K., Elshikh M.S., Al Farraj D.A.A. (2022). Property Management of BiFeO3-Based Multifunctional Perovskite Nanomaterials: Nanoparticles, Ceramics, and Thin Films. Nanomaterials for Energy Conversion, Biomedical and Environmental Applications.

[50] Yastrebov S.G., Lomanova N.A. (2021). Specific Features in the Interaction between BiFeO_3_ Nanoclusters Synthesized by Solution Combustion. Tech. Phys. Lett..

